# A Multi-Step Fusion Network for Semantic Segmentation of High-Resolution Aerial Images

**DOI:** 10.3390/s23115323

**Published:** 2023-06-03

**Authors:** Yirong Yuan, Jianyong Cui, Yawen Liu, Boyang Wu

**Affiliations:** College of Oceanography and Space Informatics, China University of Petroleum (East China), Qingdao 266580, China; 2016020124@s.upc.edu.cn (Y.Y.); 2016020102@s.upc.edu.cn (Y.L.); 2016020121@s.upc.edu.cn (B.W.)

**Keywords:** semantic segmentation, attention mechanism, multi-branch network

## Abstract

The demand for semantic segmentation of ultra-high-resolution remote sensing images is becoming increasingly stronger in various fields, posing a great challenge with concern to the accuracy requirement. Most of the existing methods process ultra-high-resolution images using downsampling or cropping, but using this approach could result in a decline in the accuracy of segmenting data, as it may cause the omission of local details or global contextual information. Some scholars have proposed the two-branch structure, but the noise introduced by the global image will interfere with the result of semantic segmentation and reduce the segmentation accuracy. Therefore, we propose a model that can achieve ultra-high-precision semantic segmentation. The model consists of a local branch, a surrounding branch, and a global branch. To achieve high precision, the model is designed with a two-level fusion mechanism. The high-resolution fine structures are captured through the local and surrounding branches in the low-level fusion process, and the global contextual information is captured from downsampled inputs in the high-level fusion process. We conducted extensive experiments and analyses using the Potsdam and Vaihingen datasets of the ISPRS. The results show that our model has extremely high precision.

## 1. Introduction

The study of remote sensing image processing technology is crucial for the area of remote sensing. The dynamic transformation information of a region, such as changes in vegetation or land use and urban changes, can be obtained from remote sensing images produced in different periods. In computer vision, one of the most significant challenges is the precise and effective segmentation of images in terms of their semantic content. It assigns categories to all pixels of the target image and labels them. It is often utilized in a variety of areas, including medical imaging [1], geographic information systems [2], remote sensing [3], and unmanned driving [4].

At an early stage, semantic segmentation was mainly implemented based on traditional image segmentation methods or random forest (RDF) [5], support vector machine (SVM) [6], and other technologies. There are two main categories of traditional image segmentation methods: edge detection-based methods and region-based methods. Image segmentation methods based on edge detection detect the target boundary points through calculation with local differential operators. Common edge detection operators include gradient operators, the Roberts operator, and the Canny edge detector. Region-based image segmentation methods perform image segmentation using the differences in attributes between the target region and the background region. Common image segmentation methods based on region include the region splitting and merging technique, as well as the region growing technique [7]. While these techniques can address the issue of discontinuous image segmentation, they have the potential to over-segment the image during the segmentation process. These methods use techniques such as RDF and SVM to extract image features and to classify pixels.

Deep learning has advanced significantly in semantic segmentation because of its rapid development. For deep-learning-based semantic segmentation, there are three typical architectures, namely, the encoder–decoder segmentation structure, the pyramid structure, and the multi-branch structure. In 2015, Long et al. advocated for the use of deep learning in semantic segmentation by proposing a full convolutional network (FCN) [8] and for using it to extend semantic segmentation to the pixel level. The U-Net model [9] was presented, which effectively integrated deep and shallow semantic information. Other examples of such networks include the PSPNet [10], SegNet [11], decoupleNet [12], and Deeplab [13]. These architectures enhance the connection between pixels by increasing the receptive field and can better obtain contextual information. However, these methods often lead to the omission of detailed targets and produce discontinuous segmentation boundaries.

In the existing network structures, deep networks prioritize the extraction of semantic information, whereas shallow networks tend to be concerned with specific information [14,15]. The crucial factor for achieving precise image segmentation is the effective fusion of both shallow and deep networks. However, the fusion methods used in the existing semantic segmentation algorithms tend to cause a loss of details [16]. The existing networks can perform semantic segmentation of a wide range of images very well, but when ultra-high-resolution images need to be segmented at the pixel level, these networks will be unable to achieve accurate segmentation due to excessively small ranges or the lack of relevant contextual information, resulting in unsatisfactory segmentation accuracy and results.

**Figure 1:**
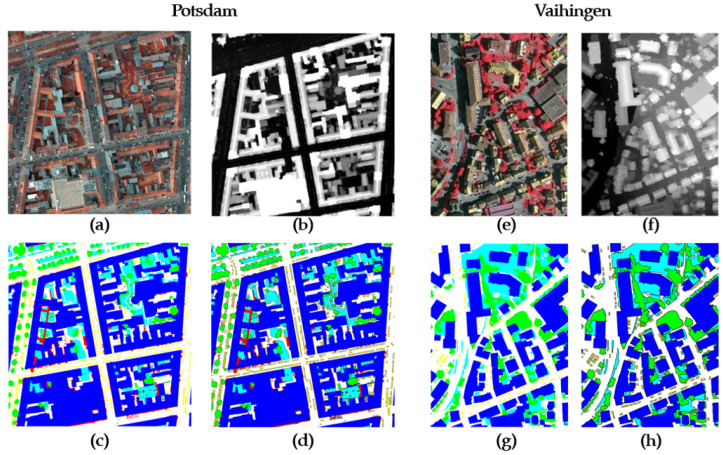
An illustrative example of the image data utilized in super high resolution. The four images on the left depict the Potsdam dataset: (**a**) near-infrared images; (**b**) digital surface model (DSM); (**c**) segmented labels; (**d**) boundary-free partition tags. The four images on the right correspond to the Vaihingen dataset: (**e**) near-infrared images; (**f**) digital surface model (DSM); (**g**) segmented labels; (**h**) boundary-free partition tags.

We propose a model that shows high accuracy in semantic segmentation of remote sensing images, particularly those representing urban environments and natural landscapes, as depicted in Figure 1.

These images include intricate information and encompass diverse scenarios encountered in daily life. The ambition of our model lies in effectuating the high-precision segmentation of such high-resolution images, thereby contributing meaningfully to applications spanning autonomous driving, urban planning, smart city development, and the construction of digital Earth representations.

The focus of this article is on presenting a fresh model for picture segmentation training that combines branches from the local, surrounding, and global areas. The new model consists of three branches that analyze the global picture after being downsampled, the local image after being cropped, and the surrounding factors in the local area. They are fused through a two-level fusion mechanism. First, the local and surrounding branches are fused. Then, the output results are fused with the input image of the global branch. This structure can effectively balance the usage of GPU memory, and most importantly, it can significantly raise semantic segmentation’s accuracy level. If very small local areas are of concern, they can be segmented precisely by the local and surrounding branches. The design of this structure enables the seamless integration of high-precision local specific and global contextual information, which is balanced by learning to maintain accurate segmentation. The main method is summarized below.

1. A high-precision multi-branch network structure is proposed for ultra-high-resolution picture semantic segmentation.
2. This structure can effectively combine the global contextual information and fine local features, introduce surrounding branches to ensure high-precision local segmentation while preserving the spatial relationship, and reduce the influence of noise to a certain extent.
3. The network is designed with a two-level fusion mechanism and uses the SENet and transformer structure to further improve the accuracy of semantic segmentation.

## 2. Related Work

### 2.1. Semantic Segmentation

An FCN [8] can adapt to the input of images of any size. It substitutes convolutional layers for all of the fully connected layers in the convolutional neural network and uses the softmax function to classify pixels and to achieve semantic segmentation at the pixel level. The U-Net [9] performs segmentation by cell superposition and improves the segmentation accuracy by connecting the feature images in the encoder and decoder. The PSPNet [10] uses a pyramid pooling layer to connect global information at different scales, and it can integrate background information very effectively with an overall accuracy of around 90%. Based on FCN, Google has proposed a series of image semantic segmentation models known as Deeplab [13]. These models can obtain image feature information based on multi-scale perception. The DeepLabV3+ [17,18] in the Deeplab series captures more contextual information by increasing the receptive field using atrous spatial pyramid pooling (ASPP [19]), and its mean f1 scores can reach 89.57. However, while the receptive field is increased, small-scale objects can be lost, resulting in discontinuous boundary segmentation. The semantic segmentation methods described above require many parameters and long computing time, and they neglect factors such as computing efficiency and memory consumption. For these reasons, their application in ultra-high-resolution image segmentation is restricted to a certain extent. Parallel asymmetric convolution modules, such as LedNet [20], DABNet [21], RegSeg [22], UHRSNet [23], and Dense2Net [24], have attracted wide attention. These modules prioritize context-based data while potentially overlooking the influence of global information on image segmentation.

### 2.2. Multi-Branch Networks

Multi-branch networks can perform calculations independently through different branches. Such networks are often used to learn multi-perspective and multi-scale information, and they can ensure the real-time performance and high efficiency of the network structure. They have been extensively utilized in computer vision [25,26]. Most of the existing two-branch networks use the combinations of “deep network and low-resolution input” and “shallow network and high-resolution input”, which can greatly reduce the computing cost. The characterization ability of a global multi-branch RNN can be improved by modeling the time delay in time series data [16]. Wang et al. [27] proposed a multi-branch network structure with joint channel attention, which is mostly made up of global branch, target, and component branches and can obtain abundant local feature information. Herzog et al. [28] introduced a multi-branch network architecture that utilizes the OSNet [29] as a foundation, which consists of global, local, top erase, and channel branches and can further extract finer features. Wu et al. [30] presented a multi-branch network structure with local modules to increase the capacity for generalization and stability of the network based on the uncertainty of channel attention.

Distinguished from the prevailing dual-branch structures, our model employs a three-level branch architecture. By incorporating both the local and surrounding branches, our approach enhances the model’s ability to capture fine-grained image details. Additionally, the global branch is utilized to establish spatial relationships, addressing the challenge of spatial contextual information loss. Furthermore, this design choice alleviates some of the computational burdens associated with high-precision image segmentation.

### 2.3. Attention Mechanism

The concept of the attention mechanism is to use computers to mimic human vision, to assign weights to different levels, to enable computers to automatically be mindful of the critical information contained in the input image, to adaptively suppress other useless information, and to efficiently minimize the noise interference brought by the background image. The attention mechanism was first used in recursive neural networks (RNNs) to encode input statements. The attention mechanism is employed to obtain feature information from feature graphs in convolutional neural networks. The channel attention module SENet [31] was suggested by Hu et al., in 2019, which uses the channel attention mechanism for learning through global pooling and highlights crucial information while de-emphasizing other information. Based on the U-Net, Yang et al. added the SE channel attention module to optimize image segmentation. Woo et al. proposed CBAM [32], which integrates both channel and temporal attention mechanisms to augment the model’s effectiveness and to capture comprehensive attention information. The ECANet [33] was designed with a local cross-channel interaction strategy, which did not require dimensionality reduction and which achieved remarkable results. For the Gsop-net [34], a GSoP module is incorporated into the backbone network to obtain high-order statistics efficiently. The CCNet [35] captures dependencies between pixels through a cross-attention module. In the area of computer vision, the transformer [36] utilized for natural language processing has also received extensive attention. Others in incremental segmentation are also involved [37,38].

Our model incorporates two attention mechanisms and achieves the fusion of features from different branches. The local and surrounding branches employ SENet to highlight detailed information and to facilitate feature map fusion. Moreover, we introduce a transformer structure with a single-head attention mechanism to capture the contextual information of the image, enabling high-precision segmentation of high-resolution images.

### 2.4. Comparison

In our proposed model, we emphasize both local and contextual information, utilizing a multi-branch network as the overarching framework. Our approach distinctively contemplates the influence of the target details’ surrounding environment on segmentation. To this end, we introduce an attention mechanism module and implement hierarchical fusion. On one side, the SENet adaptively calculates weight coefficients. By adding the results from different SENet modules directly, noise is mitigated, enabling finer segmentation of local details. On the other side, we integrate a transformer structure with global branches. Rather than employing an encoder–decoder structure or multi-head attention, we transform the transformer into a single-head attention module. Through processing the input of the multi-branch structure, a significant amount of contextual information can be captured, which bolsters segmentation accuracy.

## 3. Methods

### 3.1. Overview

**Figure 2:**
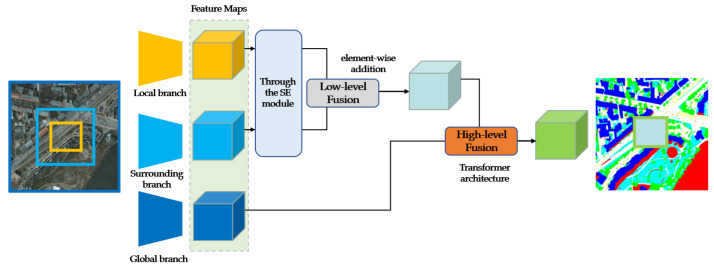
Overview of the proposed model. The local branch performs image cropping, the surrounding branch captures twice the input range of the local branch as its input, and the global branch performs downsampling. The SENet is used to achieve low-level fusion for the local and surrounding branches. Based on low-level fusion, the global branch is added for high-level fusion, and fusion is performed on the transformer structure to realize high-precision semantic segmentation.

Figure 2 illustrates the structural design of the proposed model. The input image is divided into three parts, and the local branch of the model takes a specific region of the image as its input. The region is enlarged, and its environmental image is captured and used as input to the surrounding branch, which is twice the input range of the local branch. The global image is used as input to the global branch. First, the inputs to the local and surrounding branches are fused, and the processed regional image and its surrounding image are fused through low-level fusion using the SENet [31]. Then, the output result of the low-level fusion is fused with the input image of the global branch, which is through high-level fusion based on the transformer [36]. Finally, the output result is convolved to obtain a high-precision semantic segmentation image that covers the same region as the local branch.

### 3.2. Multi-Branch

Currently, the main GPU-based segmentation methods for high-resolution images are downsampling and cropping. However, if only one of these methods is used, the segmentation boundary may be inaccurate, and segmentation may occur as a result of the loss of spatial contextual information. Some scholars [16] have experimentally demonstrated that, when the semantic segmentation of images is performed only by downsampling, details will be lost in the process of downsampling, and incorrect segmentation can occur at subtle boundaries. If semantic segmentation is performed only by cropping, when each cropped image is trained, the individual high-resolution images will lack spatial information and correlation, resulting in a large number of errors in classification.

Image segmentation methods usually use “low-resolution inputs + deep networks” or “high-resolution inputs + shallow networks”. How to realize the effective fusion of a shallow network and deep network is the key to high-precision semantic segmentation. Li et al. [14] proposed a context semantic refinement deep network to ensure the precision of segmentation. Guo et al. presented ISDNet [15], harmonizing shallow and deep network layers, and innovated a unique feature fusion module for optimized segmentation. The multi-branch network can process multi-resolution input images simultaneously, retain the detailed boundary information of high-resolution images, obtain sufficient spatial contextual information through downsampling, connect the spatial contextual information of high-resolution photographs in series, and significantly increase the precision and accuracy of segmenting high-resolution images.

Another common problem in processing high-resolution images is computational efficiency. When a single-branch network structure processes high-resolution images or multi-resolution inputs, a more complex network structure will be adopted for calculation, and a residual network, such as ResNet [39], will be used to solve the overfitting caused by the increase in network layers. However, the increased complexity of the network leads to a larger computational workload, resulting in decreased computing efficiency. This may limit its applicability in a wider range of scenarios.

Our model incorporates a three-level branch structure and two-level fusion strategy. At the local branch, we input cropped images to focus on local details. The surrounding branch extracts information around the input image for the local branch. At the global branch, we input downsampled results of the original image to capture a broader spatial context. We perform a low-level fusion between the local branch and the surrounding branch to mitigate the impact of image noise and to ensure accurate segmentation of fine details. Furthermore, we integrate the results of the low-level fusion with the global branch, thereby preserving both the global information and spatial contextual relationships in the image.

The adoption of a multi-branch network structure allows for concurrent processing, thereby mitigating the necessity of excessively complex network architectures in the feature extraction phase. This architectural design choice serves to address the computational efficiency issues that arise when dealing with ultra-high-resolution images.

### 3.3. Multi-Level Fusion

#### 3.3.1. Low-Level Fusion

**Figure 3:**
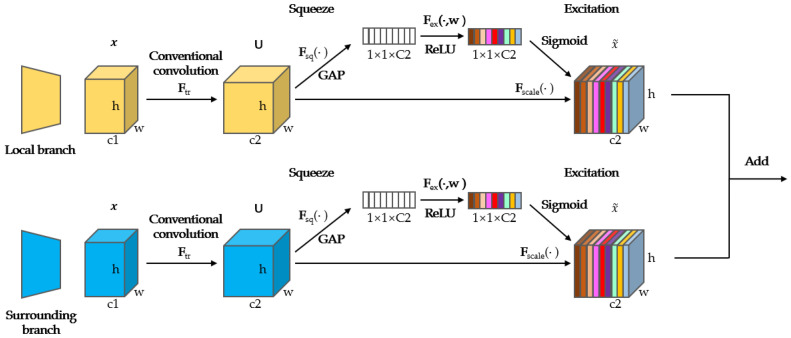
Detailed process of low-level fusion. The SENet is used as the general architecture. The input images of the local and surrounding branches are compressed using traditional convolution and global average pooling and are processed through fully connected layers after the weight of each channel is assigned. Unlike the SENet, our model adds and fuses the output results of the local and surrounding branches after processing through fully connected layers to obtain a new feature image.

Figure 3 depicts the precise steps of low-level fusion. Low-level fusion is used to fuse the inputs to the local and surrounding branches. The local branch obtains the semantic information of the image, and the surrounding branch mitigates the global images’ noise while retaining the local branch’s contextual information, thus obtaining accurate image information. The fusion of low-level features of images is mainly carried out based on the SENet. An important advantage of the SENet is that it can pay attention to the relationship between different channels. It can automatically enable the determination of varying feature significance across channels, calculate weighting coefficients, assign weights to images, and thereby obtain more image information and improve the accuracy of image semantic segmentation.

We utilize two metrics, namely top-1 error and top-5 error, to showcase the exceptional performance of SENet in semantic segmentation. The top-1 error refers to comparing the model’s classification prediction for each sample with the actual label. The prediction is considered correct if the model’s prediction matches the highest probability category in the real label. The top-5 error involves comparing the model’s prediction with the actual label and checking if the model’s prediction includes the category of the actual label. The specific comparison results are presented in Table 1.

For this network, both the local branch and surrounding branch are independently inputted, each with a spatial dimension of *w* × *h*. These input elements undergo convolution using conventional methods, transforming the number of feature channels from *c*1 to *c*2. The function is defined in Equation (1).

(1) $$u_{c}=V_{c}\times X=\sum_{s=1}^{c^{\prime }}{V_{c}}^{s}\times X^{s}$$

The equation is a standard convolution operation. *U* represents that there are *C* feature maps, each with a size of h×w. $U_{c}$ denotes the *c*th channel of the input *U*, while $V_{c}$ represents the *c*th convolution kernel, and $X^{s}$ refers to the *s*th input.

The image features are squeezed by means of global average pooling. The squeeze function is defined in Equation (2).

(2) $$F_{sq}\left(u_{c}\right)=\frac{1}{H\times X}\sum_{i=1}^{H}\sum_{j=1}^{W},u_{c}\left(i,j\right)$$

where $F_{sq}$ represents the compression function, $U_{c}$ denotes the *c*th channel of the input *U*, and *H* and *W* represent the height and width of the input, respectively.

Each two-dimensional feature channel is represented by a real integer with a certain global receptive field, and an output dimension consistent with the count of feature channels for input is maintained. At this time, the channel descriptor we obtain after the global space information is squeezed has a length of 1 × 1 × c2. Subsequently, an excitation operation is conducted. Through utilization of the ReLU activation function and the training parameter w, we can derive the weight value corresponding to the input channel, and the feature information of important channels can be obtained after the weight is assigned to the image itself. This process utilizes two fully connected layers to decrease the computational load by narrowing channels and maintaining the quantity of output channels consistent with the input. Ultimately, the output weight acquired from the activation operation is reapplied through the sigmoid function, enabling the capture of more intricate inter-channel dependencies. The excitation function is defined in Equation (3).

(3) $$F_{ex}\left(z,W\right)=\sigma \left(W_{2}\delta \left(W_{1}z\right)\right)$$

where $F_{ex}$ represents the excitation function, and *z* is the input signal squeezed from the previous layer. δ denotes the ReLU activation function. The dimension of $W_{1}$ is $\frac{c}{r}\times c$, where r is a scaling parameter. This parameter aims to decrease the number of channels, thereby reducing the computational workload. The output is then multiplied by W2, which represents a fully connected layer operation. The dimension of $W_{2}$ is $c\times \frac{c}{r}$, resulting in an output dimension of 1×1×c. Finally, the output is obtained by applying the sigmoid function.

By implementing channel-wise multiplication, these weights are imposed onto the preceding features, thereby accomplishing the recalibration of the original features, as shown in Equation (4).

(4) $$F_{scale}\left(U_{c},S_{c}\right)=S_{c}\cdot U_{c}$$

The results obtained by the two branches are the local and the surrounding branches with weight characteristics. After applying the SE module, both feature maps possess identical feature dimensions. Consequently, a straightforward summation fusion operation is employed to integrate the element-wise addition of these two feature results, receiving an outcome for the low-level fusion.

#### 3.3.2. High-Level Fusion

**Figure 4:**
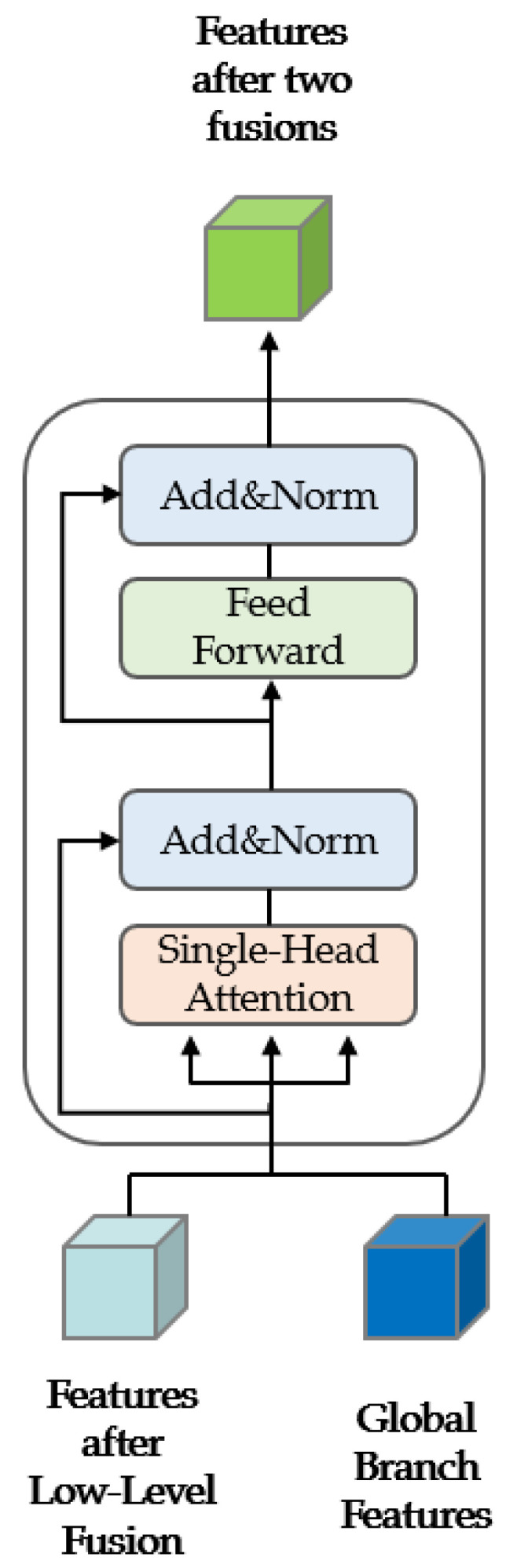
Detailed process of high-level fusion. Unlike the self-attention mechanism in the transformer, our model uses the output features obtained by low-level fusion and the input images of the global branch as the inputs for high-level fusion, and it obtains the output results of high-level fusion after the attention calculation and processing with fully connected layers.

The precise steps of high-level fusion are depicted in Figure 4. The transformer architecture exhibits an exceptional capability for comprehending the global context within an image. It achieves this by segmenting the image into a series of patches and by computing attention scores among these patches. This method enables the model to attend to all patches associated with the current patch. This innovative approach effectively addresses local segmentation inaccuracies that can arise when attempting to capture global information.

The transformer structure was used as the structure for high-level fusion in our model, and the multi-head attention mechanism was simplified into a single-head attention mechanism. In a transformer model, each “head” operates as an independent attention mechanism, computing attention scores autonomously. In our study, we reconfigure the model by setting the number of these “heads” to one, subsequently resizing the model dimensions correspondingly. This allows for the complete dimensionality of the input embedding to be processed by a singular attention head.

Then, three attention calculations were performed, the output results of low-level fusion were fused with the global images of the global branch through high-level fusion, and accurate results of semantic segmentation of ultra-high-resolution images were achieved.

**Figure 5:**
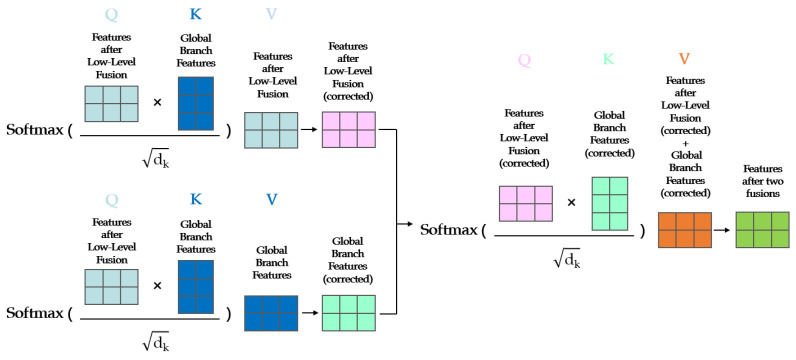
Attention calculation process. The features after low-level fusion and the input image of the global branch are calculated twice by softmax, and the output results are calculated again by softmax to obtain the output results of high-level fusion. In the equation, $\sqrt{d_{k}}$ signifies the number of columns in the Q and K matrices, which correspond to the dimension of the vector. To prevent the dot product from becoming excessively large, the result is divided by the square root of $\sqrt{d_{k}}$.

The attention calculation process is shown in Figure 5. The attention value was calculated three times in the proposed network structure. In the computation of the attention value, the choice of query, key, and value, denoted as Q, K, and V, respectively, is crucial. Q embodies the context of the current segment, whereas K serves to distinguish whether the information is pertinent to this context, and V represents the actual content, which is the specific information we aim to extract from the data.

During the calculation of the attention value, Q, K, and V were replaced with our input images, namely, the features after low-level fusion and the global images input into the global branch. Within this process, the features resulting from low-level fusion are employed as Q, while the input from the global branch is used as K. The dot product of these two is computed, after which the low-level fused features and the input from the global branch are each utilized as V to perform two attention computations, effectively achieving two instances of self-fusion. The result of the dot product computation represents similarity, and a higher value corresponds to greater similarity and a heightened degree of attention. For general images, low-level features include edge and texture features, and global represents high-level features, i.e., category features. High-level fusion was carried out after the features in the two layers were placed in the same dimensional space through the self-fusion operation. The benefit of this method is that it can give the information regarding the intrinsic features more consideration and can prevent the final result from being completely dominated by a single feature due to the inconsistency of the dimensionality of the feature space.

After self-fusion is completed, the two features are taken as Q and K, and the sum of these two features is taken as V. The third attention calculation is carried out to obtain the accurate semantic segmentation results of ultra-high-resolution images.

## 4. Experiments

### 4.1. Details

#### 4.1.1. Data Set

In this paper, the Vaihingen and Potsdam datasets are used. Images for both datasets [43] were taken using a digital aerial camera of the German Association for Photogrammetry and Remote Sensing (DGPF) [44] and Mosaic using Trimble INPHO OrthoVista. The two datasets consist of images falling into six categories, including roads, buildings, low-growing plants, trees, cars, and backgrounds, which are widely used in the field of remote sensing image recognition.

The Vaihingen dataset is a collection of aerial images of a town in Germany with a high-resolution orthophoto, digital surface model (DSM), and accurate ground truth data for urban areas. It consists of 33 aerial images of different sizes, and 16 of them have labels. Each image contains an average of 2494 × 2064 pixels. The six categories of images in this dataset are unevenly distributed. The pixel count of each category is shown in Table 2.

The Potsdam dataset is a collection of typical urban scenes with large buildings, tiny streets, and dense structures in the settlement. This dataset is unique due to its combination of orthophoto imagery and DSM derived from LiDAR data, offering a composite view of the urban landscape. This intricate detail provides a robust foundation for advanced research, especially in fields such as machine learning and artificial intelligence for object detection, semantic segmentation, and change detection in urban areas. This dataset contains 38 aerial images, 24 of which have been labeled. All images in it have the same size of 6000 × 6000 pixels. The six categories of images in this dataset are also unevenly distributed. The pixel count of each class in the dataset is depicted in Table 2.

#### 4.1.2. Evaluation Index

Based on the overall accuracy (OA), F1 score (F1) and mean intersection over union (mIoU) are utilized as evaluation indices.

F1: The harmonic mean of both precision and recall is defined in Equation (5)

(5) $$F1=\frac{TP}{2\times TP+FN+FP}$$

OA: Predict the ratio of the correct pixel value to the total pixel value, as shown in Equation (6)

(6) $$OA=\frac{TP+TN}{TP+FN+FP+TN}$$

mIoU: Add up the IDs of each category and then divide by the total number of categories, as shown in Equation (7).

(7) $$mIoU=\frac{1}{k+1}\sum_{i=0}^{k}\frac{TP}{FN+FP+TP}$$

In the above formula, TP denotes instances where the model accurately forecasts a positive result. FP represents situations where the model predicts a positive outcome erroneously, as the actual result is negative. FN arises when the model incorrectly foresees a negative outcome while the true result is positive. TN signifies cases in which the model correctly anticipates a negative outcome.

#### 4.1.3. Experimental Details

Based on the existing literature [45,46], in the Vaihingen dataset, we followed the benchmark organizer’s recommendation of utilizing 16 images for training and 17 images for testing. Similarly, in the Potsdam dataset, we employed a training set consisting of 24 tiles and a testing set comprising 14 tiles.

For our experiments, the basis of the network is an FPN (feature pyramid network) with ResNet101. The feature map sharing technique was used for the top–down ResNet101 feature maps from conv2 to conv5 and for smooth phases of the FPN. Both the local picture that was cropped and the downsampled global image are 500 × 500 pixels in size. An overlap of 50 pixels between adjacent patches was set to avoid the problem of vanishing boundaries for all convolutional layers. We utilized the command-line utility “gpustat”, set the batch size to 1, and avoided gradient computation to assess the GPU memory usage of the model. All research was completed using workstations with NVIDIA 1080Ti GPUs, and only one GPU was utilized for training and reasoning. Our experimental framework is based on PyTorch, and the Adam optimizer was used. The global branch is trained at a learning rate of 1 × $10^{-4}$, while the local and surrounding branches are trained at a learning rate of 2 × $10^{-5}$. The learning rates associated with the global, local, and surrounding branches are determined through an exhaustive process of parameter adjustment. This method ensures the achievement of an optimal training velocity and superior end performance for the model. During the experiments, a small batch size of six was used to be trained.

### 4.2. Result

Table 3 and Table 4 include a list of the experimental findings for the Vaihingen and Potsdam datasets. The quantitative index verifies the validity of our model. Specifically, the OA and mIoU results produced by the proposed model are 92.40% and 84.43% for the Vaihingen dataset and 92.36% and 87.73% for the Potsdam dataset, which are significantly better than those of most ResNet-based methods. Our model is far superior to current contextual information fusion techniques such as DeepLab V3+ and PSP Net, and the average OA and mIoU results of our model are 1.54% and 2.77% higher than those of the aggregation methods mentioned above. Meanwhile, our approach outperforms some multi-scale feature fusion models, such as the EaNet, especially in terms of the recognition of buildings and low vegetation. The results concerning semantic segmentation of the images of buildings and low vegetation are 98.90% and 89.32%, which have been improved by 3.15% and 4.87%, respectively. Our model’s overall accuracy is higher than that of transformer neural networks such as the BoTNet, and the OA and mIoU results of our model are 1.74% and 2.75% higher than those of transformer neural networks. However, for the recognition of certain types of ground objects such as trees, the accuracy of our model is similar to that of other models compared with it. In general, our model’s accuracy is significantly better than that of other models.

When the target information is situated at the image periphery, our model’s segmentation accuracy may experience a slight reduction. However, this is primarily in comparison to the central regions of the image and is generally within acceptable margins. To mitigate the impact of image edges on semantic segmentation accuracy, we implement padding at the image periphery. This helps to reduce the influence of image edges on semantic segmentation accuracy.

We stipulate that an image is an edge region if it contains anything else within seven pixels of it. By conducting experiments on the Vaihingen dataset and the Potsdam dataset, we obtained comparative results, as shown in Table 5. In the Vaihingen dataset, the mIoU for the edge of the image is 62.40, while the mIoU for the central region of the image is 89.17. In the Potsdam dataset, the values were 76.34 and 89.85.

In terms of computational capacity, we measure it using Floating Point Operations (FLOP). Compared to the concatenated transformer structure, our network achieves a FLOP of 451.2, while the concatenated transformer structure requires 824.7 FLOP. This reduction in computational resources helps alleviate constraints on the network while also reducing computation time. Furthermore, our model is not significantly affected by the large volume of data during the training process. Compared to the concatenated transformer structure, our model’s training time is merely two-thirds of its duration.

Our model endeavors to accomplish high-precision semantic segmentation of ultra-high-resolution remote sensing images. To reach such elevated levels of accuracy, trade-offs are inevitably necessitated, particularly with computational complexity. Given the intricate computations involved in processing the extensive information embedded within ultra-high-resolution images, it is both understandable and acceptable that our model demands a measure of computational power and processing time. Consequently, the performance of the model might be curtailed under circumstances where computational resources are limited, or where rapid image processing is indispensable. Furthermore, opportunities for future exploration and enhancement lie in potential efficiency improvements within our model.

### 4.3. Ablation Study

We propose a model that utilizes two-level fusion to improve image segmentation performance. Therefore, it is worthwhile to carry out an ablation study and look into how each model component affects precision, the experimental results are shown in Table 6. First, ResNet101 was selected for the ablation study. From the results for the two datasets, it is evident that the mIoU findings obtained by the ResNet101 alone are lower. Then, low-level fusion was performed, and the local and surrounding features were fused through the SENet structure. The results show that only low-level fusion has greatly improved the mean F1 by 3.48% on average, and it has a positive effect on OA and has greatly improved the mIoU by 4.99% on average. When the transformer structure is used to fuse the features after low-level fusion, and for the global branch, with high-level fusion only, the model’s mean F1 and mIoU performance shows a slight improvement compared to low-level fusion. However, the OA results are similar to those obtained by low-level fusion. Finally, the low-level and high-level fusion processes were combined. The SENet was used to improve accuracy in local areas, and then, the transformer was used to connect the contextual information. The multi-level fusion approach outperforms the single low-level or high-level fusion methods in terms of both OA and mIoU, which were 3.02% and 7.43% higher than those obtained by the ResNet101.

The ablation study indicates that both the low-level and high-level fusion processes are essential and cannot be removed without degrading the performance of the model, and accurate semantic segmentation can be achieved only when these two fusion processes are combined to process images.

## 5. Conclusions and Future Work

In the task of semantic segmentation for high-resolution images, achieving accurate segmentation results necessitates a comprehensive analysis of both detailed features and contextual information within the image. This paper presents a novel model specifically designed for performing ultra-high-resolution remote sensing imagery’s semantic segmentation. This model uses an attention mechanism and a multi-branch structure to perform feature fusion at two levels, and by adding the SENet module and transformer, it can effectively complete fine image processing through the local and surrounding branches and enhance the precision of semantic segmentation using the global contextual information captured by the global branch. We conducted extensive testing on the Vaihingen and Potsdam datasets, which encompass a diverse range of urban scenes and natural landscapes, ensuring the robustness of our approach. Compared to the majority of ResNet-based models, this model has higher segmentation precision.

Our research holds immense practical significance, as it offers valuable insights that can be applied across various domains in real-world applications. In fields such as unmanned driving and smart city, accurate semantic segmentation will greatly improve the security of targets to be identified or the accuracy of information systems, and it has significant real implications. Additionally, our approach enables the processing of remote sensing images within a specific time frame, allowing for the extraction of intricate information. This enables us to discern the patterns of development and changes in ground objects within a designated area, thus providing essential decision support for relevant stakeholders.

Our future work will focus on enhancing computational efficiency and promoting model lightweights. We aim to optimize the model architecture to reduce computational burdens while maintaining accuracy. This includes utilizing techniques such as deep convolution and point convolution instead of standard convolution, as well as integrating void convolution into the SE module to minimize computational parameters. Additionally, we will adjust the multi-branch structure and introduce a dynamic weight adjustment mechanism to accelerate processing speed without significantly impacting performance. 

## Figures and Tables

**Table 1:** Comparison of error rates between SENet and other networks on 224 × 224 and 320 × 320 clipped images.

Methods	Top-1 Error 224×224	Top-5 Error 224×224	Top-1 Error 320×320	Top-5 Error 320×320
ResNet-152 [39]	23.0	6.7	21.3	5.5
ResNet-200 [40]	21.7	5.8	20.1	4.8
Inception-v4 [41]	-	-	20.0	5.0
DenseNet [42]	22.15	6.12	-	-
SENet	18.68	4.47	17.28	3.79

**Table 2:** The proportion of pixels in the Vaihingen and Potsdam datasets for each category.

Data Set	Roads	Buildings	Low Plants	Trees	Cars	Backgrounds
Vaihingen	27.94%	26.15%	20.84%	23.19%	1.21%	0.67%
Potsdam	28.46%	26.72%	23.54%	14.64%	1.69%	4.96%

**Table 3:** Results of experiments on the Vaihingen dataset.

Methods	Backbone	Roads	Buildings	Low Plants	Tree	Cars	Mean F1	OA	mIoU
DeepLab V3+ [17,18]	ResNet 101	92.38	95.17	84.29	89.52	86.47	89.57	90.56	81.47
PSP Net [10]	ResNet 101	92.79	95.46	84.51	89.94	88.61	90.26	90.85	82.58
DANet [47]	ResNet 101	91.63	95.02	83.25	88.87	87.16	89.19	90.44	81.32
EaNet [48]	ResNet 101	93.40	96.20	85.60	90.50	88.30	90.80	91.20	-
DDCM-Net [49]	ResNet 50	92.70	95.30	83.30	89.40	88.30	89.80	90.40	-
CASIA2 [46]	ResNet 101	93.20	96.00	84.70	89.90	86.70	90.10	91.10	-
V-FuseNet [45]	FuseNet	91.00	94.40	84.50	89.90	86.30	89.20	90.00	-
DLR_9 [50]	-	92.40	95.20	83.90	89.90	81.20	88.50	90.30	-
BoTNet [51]	ResNet 50	92.24	95.28	83.88	89.99	85.47	89.37	90.51	81.05
Ours	ResNet 101	95.61	98.90	89.32	89.94	89.60	92.67	92.40	84.43

**Table 4:** Results of experiments on the Potsdam dataset.

Methods	Backbone	Roads	Buildings	Low Plants	Tree	Cars	Mean Fl	OA	mIoU
DeepLab V3+ [17,18]	ResNet 101	92.95	95.88	87.62	88.15	96.02	92.12	90.88	84.32
PSP Net [10]	ResNet l 01	93.36	96.97	87.75	88.50	95.42	92.40	91.08	84.88
DDCM-Net [49]	ResNet 50	92.90	96.90	87.70	89.40	94.90	92.30	90.80	-
CCNet [35]	ResNet 101	93.58	96.77	86.87	88.59	96.24	92.41	91.47	85.65
AMA_1	-	93.40	96.80	87.70	88.80	96.00	92.54	91.20	-
SWJ_ 2	ResNet 101	94.40	97.40	87.80	87.60	94.70	92.38	91.70	-
V-F useNet [45]	FuseNet	92.70	96.30	87.30	88.50	95.40	92.04	90.60	-
DST_5 [52]	FCN	92.50	96.40	86.70	88.00	94.70	91.66	90.30	-
BoTNet [51]	ResNet 50	93.13	96.37	87.31	88.01	95.79	92.12	90.76	85.62
Ours	ResNet 101	94.50	97.49	88.47	91.0	97.10	93.71	92.36	87.73

**Table 5:** Performance comparison of image edge and image center in the dataset.

Dataset	Image Region	mIoU
Vaihingen	Image center	89.17
Vaihingen	Image edge	62.40
Potsdam	Image center	89.85
Potsdam	Image edge	76.34

**Table 6:** The Vaihingen and Potsdam datasets’ ablation study.

Dataset	Methods	Mean F1	OA	mIoU
Vaihingen	ResNet101	85.10	89.49	75.48
Vaihingen	ResNet101 + LF	88.96	90.73	80.48
Vaihingen	ResNet101 + HF	89.48	90.87	81.26
Vaihingen	ResNet101 + LF + HF	92.67	92.40	84.43
Potsdam	ResNet101	88.66	89.24	79.97
Potsdam	ResNet101 + LF	91.75	90.45	84.95
Potsdam	ResNet101 + HF	91.61	90.59	85.21
Potsdam	ResNet101 + LF + HF	93.71	92.36	85.87

## Data Availability

Not applicable.
